# Synergistic regulation of uterine radial artery adaptation to pregnancy by paracrine and hemodynamic factors

**DOI:** 10.1152/ajpheart.00205.2023

**Published:** 2023-08-04

**Authors:** H. H. Allerkamp, S. Leighton, T. Pole, A. R. Clark, J. L. James

**Affiliations:** ^1^Department of Obstetrics and Gynecology, Faculty of Medical and Health Sciences, University of Auckland, Auckland, New Zealand; ^2^Auckland Bioengineering Institute, University of Auckland, Auckland, New Zealand

**Keywords:** computational model, pregnancy, shear stress, uterine radial arteries, vascular adaptation

## Abstract

Fetal growth throughout pregnancy relies on delivery of an increasing volume of maternal blood to the placenta. To facilitate this, the uterine vascular network adapts structurally and functionally, resulting in wider blood vessels with decreased flow-mediated reactivity. Impaired remodeling of the rate-limiting uterine radial arteries has been associated with fetal growth restriction. However, the mechanisms underlying normal or pathological radial artery remodeling are poorly understood. Here, we used pressure myography to determine the roles of hemodynamic (resistance, flow rate, shear stress) and paracrine [β-estradiol, progesterone, placental growth factor (PlGF), vascular endothelial growth factor] factors on rat radial artery reactivity. We show that β-estradiol, progesterone, and PlGF attenuate flow-mediated constriction of radial arteries from nonpregnant rats, allowing them to withstand higher flow rates in a similar manner to pregnant vessels. This effect was partly mediated by nitric oxide (NO) production. To better understand how the combination of paracrine factors and shear stress may impact human radial artery remodeling in the first half of gestation, computational models of uterine hemodynamics, incorporating physiological parameters for trophoblast plugging and spiral artery remodeling, were used to predict shear stress in the upstream radial arteries across the first half of pregnancy. Human microvascular endothelial cells subjected to these predicted shear stresses demonstrated higher NO production when paracrine factors were added. This suggests that synergistic effects of paracrine and hemodynamic factors induce uterine vascular remodeling and that alterations in this balance could impair radial artery adaptation, limiting blood flow to the placenta and negatively impacting fetal growth.

**NEW & NOTEWORTHY** Placenta-specific paracrine factors β-estradiol, progesterone, and placental growth factor attenuate flow-mediated constriction of the rate-limiting uterine radial arteries, enabling higher flow rates in pregnancy. These paracrine factors induce their actions in part via nitric oxide mediated mechanisms. A synergistic combination of paracrine factors and shear stress is likely necessary to produce sufficient levels of nitric oxide during early human pregnancy to trigger adequate uterine vascular adaptation.

## Graphical abstract

**Figure:**
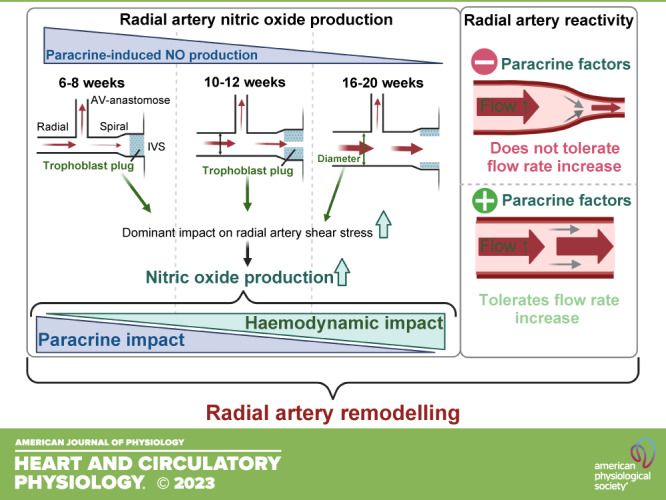

## INTRODUCTION

A healthy pregnancy depends on the successful establishment of the placenta. This transient fetal exchange organ lays the foundation for fetal nourishment in utero, facilitating oxygen and nutrient transfer between the maternal and fetal circulations. To enable this, the uterine blood vessels adapt structurally and functionally to pregnancy by becoming wider and more compliant, ultimately delivering a 15-fold increase in blood flow to the placenta (1–4). Impaired uterine vascular adaptation can lead to insufficient delivery of oxygen and nutrients to the fetus, severely limiting its growth [fetal growth restriction (FGR), ≤3rd growth percentile] (5). Although the pathophysiological origins of FGR arise in inadequate placental development and uterine adaptation in the first half of pregnancy, FGR cannot be clinically diagnosed until later in gestation (6). Improved understanding of the underlying mechanisms of uterine vascular adaptation to pregnancy and the spatial and temporal changes of the most relevant vessel metrics are key to better predict and detect FGR and to improve clinical outcomes.

In humans, maternal blood is supplied to the uterus via one of two uterine arteries, which extend up opposite sides of the organ. Arcuate arteries branch off each uterine artery, running circumferentially within the muscular wall of the uterus (the myometrium). Approximately 50 radial arteries branch perpendicular from each arcuate artery, extending toward the uterine lumen. All of these myometrial vessels roughly double in diameter across the first half of pregnancy via the process of outward hypertrophic remodeling (7, 8). In the inner half to third of the myometrium, arteriovenous anastomoses provide an alternate pathway for blood flow directly to the venous system, allowing a bypass in case of high downstream resistance (9), and this has important functional consequences on flow in the uterine circulation (10). Finally, within the decidua, the terminal part of the uterine vascular network is formed by ∼50–100 spiral arteries (11), which undergo dramatic change in pregnancy, transforming 4- to 10-fold in diameter from tight coils into wide funnels that open directly into the intervillous space (7, 12). Importantly, by the end of the first trimester, the spiral arteries become wider than the upstream radial arteries (7, 13), making the radial arteries key rate-limiting vessels of volumetric blood flow within the network (10, 14).

Although remodeling of the spiral arteries is facilitated by invading placental cells (extravillous trophoblasts), these cells do not reach the upstream vessels (radial, arcuate, or uterine arteries) (15, 16). Rather, it has been hypothesized that the remodeling of these larger vessels is triggered by a combination of hemodynamic (resistance, flow rate, and shear stress) and paracrine factors (2, 3, 17). Importantly, in early pregnancy, resistance, flow rates, and shear stress are determined by not only the vessel dimensions but also the extravillous trophoblasts that build plugs within the spiral arteries, which progressively break down until midpregnancy (7, 14). As the plugs break down, decreased resistance leads to an increase in flow and shear stress throughout the system, inducing endothelial production of nitric oxide (NO), which may impact vascular remodeling and compliance (3).

In addition to hemodynamic changes, hormones and paracrine factors can impact vascular function, with estrogen, progesterone, placental growth factor (PlGF), and vascular endothelial growth factor (VEGF) all shown to affect endothelial NO production or endothelial nitric oxide synthase (eNOS) activity (18–23). All of these factors increase in maternal serum across pregnancy (24–26), primarily as a result of placental production (26, 27). Importantly, such factors in the uterine microenvironment have dominant impacts on vascular adaptation compared with systemic factors (28, 29). Indeed, although steroid hormones can initiate circumferential remodeling of uterine arteries, continuation of this process depends on local paracrine factors (30, 31). Accordingly, in rats, estrogen enhances VEGF-induced uterine artery dilation (32), and both human and rat uterine arteries dilate in response to PlGF, with the latter being more sensitive to PlGF in pregnancy (20). However, studies examining how paracrine factors impact remodeling of the rate-limiting radial arteries are lacking.

Recently, we quantified changes in artery size and trophoblast plugging in the uterine vasculature across the first half of human pregnancy (7). Furthermore, we showed that the biomechanical properties and vascular reactivity to pressure and flow in rat uterine radial arteries change in pregnancy and developed a computational model to describe this uterine-specific vascular function in the nonpregnant and pregnant states (1). Although this characterized the extent of structural and functional changes, the underlying mechanisms triggering these changes remain unclear. Therefore, this study aimed to investigate the influence of pregnancy-specific paracrine factors on the flow and shear stress response of rat radial arteries and human endothelial cells.

## METHODS

### Animals and Tissues

Sprague–Dawley rats (12 to 16 wk old), either virgin and in estrus (*n* = 35, termed nonpregnant) or near term (E19.5; *n* = 6, termed pregnant), were used according to protocols approved by the University of Auckland Animals Ethics Committee (Protocol No. R2229). Animals were housed under standard conditions, and estrus was confirmed by vaginal smear. Dams were euthanized by CO_2_ and cervical dislocation, or for unborn fetuses by decapitation. Uteri were harvested immediately after euthanasia and stored on ice in physiological saline solution (PSS).

### Immunohistochemistry

Uterine and adjacent tissue from pregnant (E19.5) or nonpregnant rats (*n* = 5/group) containing an arcuate artery and radial arteries in the mesometrium was dissected and fixed in 4% paraformaldehyde. Fixed tissues were paraffin embedded and cut into 5-µm-thick serial sections using a Leica RM2245 microtome (Leica Microsystems, Germany). One slide from each series was stained with modified Miller’s elastin stain (R. A. Lamb, UK) as previously described (33) to distinguish radial arteries from veins. Slides were deparaffinised and rehydrated, and antigen retrieval was performed with sodium citrate buffer (pH 6.0) for 1 h in a pressure cooker. After being blocked with 3% H_2_O_2_ and 1% normal horse serum, slides were incubated with the following primary antibodies, diluted in 1% bovine serum albumin (BSA) overnight at 4°C in a humidity chamber: estrogen receptor (ER)α (1 µg/mL, MA5-13304 mouse monoclonal, Thermo Fisher Scientific; RRID:AB_11002193), progesterone receptor (PR; 1 µg/mL, ab2765 mouse monoclonal, Abcam, UK; RRID:AB_2164316), vascular endothelial growth factor receptor (VEGFR)-1 (0.02 µg/mL, ab32152 rabbit monoclonal, Abcam; RRID:AB_778798), and VEGFR-2 (1 µg/mL, PA5-16487 rabbit polyclonal, Thermo Fisher Scientific; RRID:AB_10978670). Antibodies were detected with the R.T.U. VECTASTAIN Universal Quick Kit (Vector) and AEC (3-amino-9-ethylcarbazole), and slides were counterstained with hematoxylin. For each antibody, sections of rat placenta were used as positive controls. Irrelevant IgG isotype-matched antibodies were used in place of primary antibodies as negative controls. ImageJ (v1.53c, NIH) was used to manually segment all radial artery layers (tunica intima, media, and adventitia) in each sample and to assess the staining intensity per cell using the color deconvolution plug-in as previously described (34).

### Pressure Myography

Uterine radial arteries were identified anatomically as branching from an arcuate artery and running toward the uterine lumen (in nonpregnant animals) or feeding into spiral arteries and running toward a placenta (in pregnant animals). Radial arteries were dissected out, cleaned, and mounted onto two glass cannulas in a pressure myograph system (114P Pressure Myograph System with FlowMeter 162FM, DMT, Denmark). Vessel diameters and flows were tracked with MyoVIEW software (DMT) as previously described (1). Briefly, each vessel underwent an adaptation period in a PSS organ bath at 37°C, reaching 50 mmHg intraluminal pressure (equivalent to in vivo pressure) followed by a standard wake-up protocol testing the vasoconstrictive response (high K^+^-PSS and 10 µM norepinephrine) and endothelium-dependent vasodilation (10 µM acetylcholine after 50% preconstriction) (1). Vessels showing <70% dilation were excluded.

These experiments sought to treat nonpregnant radial arteries with paracrine factors to determine if they would react more similarly to vessels from pregnant animals. Untreated nonpregnant (*n* = 8 radial arteries from *n* = 6 animals) and pregnant (*n* = 10 radial arteries from *n* = 6 animals) control group data were generated as part of a previous data set (1) and were used here as comparative reference values. An additional *n* = 5 or 6 radial arteries from *n* = 5 or 6 nonpregnant animals were used in this work for each treatment group. After confirmation of vessel functionality with the wake-up protocol, each radial artery was incubated in the organ bath for 20 min with either a single paracrine factor or a combination of all paracrine factors, by adding the factors directly into the organ bath in the respective concentration (Table 1). This was done under 50 mmHg intraluminal pressure but before starting flow. As no data are available on myometrial tissue concentrations of any of the paracrine factors assessed, physiologically relevant concentrations were informed by myography dose-response curves reported in the literature (Table 1). Across the experimental paradigm, all vessels then underwent stepwise increases in intraluminal flow created by increasing the difference between inlet and outlet pressure by 10 mmHg every 5 min, thereby keeping the intraluminal pressure at 50 mmHg throughout. Resulting flow rates covered a range informed by computational predictions for the uterine radial arteries of nonpregnant and pregnant rats (1). Afterward, each vessel underwent a washout period at 50 mmHg intraluminal pressure under no-flow where the solution in the organ bath was replaced with fresh PSS without paracrine factors five to six times over 15–20 min to remove paracrine factors until the vessel diameter went back to baseline. Finally, vessels were incubated for 20 min with eNOS antagonist *N*^ω^-nitro-l-arginine methyl ester (l-NAME; 0.3 mM) and then for a further 20 min with the respective paracrine factor, before the same sequence of increasing flow steps was repeated as aforemetioned. Vessels that developed any disruptions across the experimental period such as leakage were excluded.

**Table 1.** Paracrine factors and concentrations used for pressure myography and shear stress assays

Paracrine Factor	Manufacturer	Product Code	Concentration in Bath*/Media†	Reference(s)
Pressure myography experiments				
β-Estradiol	Sigma-Aldrich	E4389	3 µM*	35
Progesterone	Sigma-Aldrich	P7556	100 µM*	36
Recombinant rat				
PlGF protein	Abcam, UK	ab190989	0.1 nM*	20
VEGF_164_ protein	Sigma-Aldrich	V3638	3 nM*	32
In vitro shear stress assays				
β-Estradiol	Sigma-Aldrich	E4389	36.7 nM†	24, 37
Progesterone	Sigma-Aldrich	P7556	159 nM†	24
Recombinant human				
PlGF protein	PeproTech	100-06	6.73 pM†	25, 26

PlGF, placental growth factor; VEGF, vascular endothelial growth factor.

### Western Blot Analysis

Radial arteries were identified and sampled as described for pressure myography experiments. Cleaned radial arteries were collected in RIPA lysis buffer on ice for 30 min and centrifuged. The pellet and supernatant were separated, snap frozen, and stored at −80°C until further use. Samples from *n* = 3 rats in the same reproductive state (virgin in estrus, or pregnant, ∼45 radial arteries per group) were pooled on a microscope glass slide over ice, minced with a scalpel, resuspended in the pooled supernatant, and homogenized in an ultrasonic bath. Protein was further concentrated by spinning through Amicon ultra-0.5 centrifugal filter units (Merck, Germany) for 5 min at 14,000 *g*. Protein concentrations were quantified using the Pierce bicinchoninic acid (BCA) Protein Assay Kit (Thermo Fisher Scientific) as per the manufacturer’s instructions. Placentas were collected for use as positive controls and were digested in a similar manner, but the harvested protein did not require further concentration. Protein (10 µg) in reducing loading buffer was loaded into a 10% acrylamide SDS-PAGE for separation by electrophoresis (*n* = 3 pools for both pregnant and nonpregnant groups (assayed in technical duplicates), where each pool contained vessels harvested from *n* = 3 rats, resulting in a total of 9 rats per group. Proteins were transferred onto a nitrocellulose membrane, blocked with 3% BSA, then incubated with a primary antibody detecting eNOS (0.02 µg/mL, ab76198 monoclonal mouse, Abcam; RRID: AB_1310183) diluted in 3% BSA overnight on a rocking platform. The antibody was detected with 0.14 µg/mL of biotinylated anti-mouse IgG secondary antibody (Cat. No. 115065071, Jackson ImmunoResearch; RRID:AB_2338564) and then horseradish peroxidase-streptavidin, according to the manufacturer’s instructions (Jackson ImmunoResearch; RRID:AB_2337238). For signal detection, Amersham ECL Prime Western Blotting Detection Reagent (Cytiva) was used in conjunction with a Bio-Rad ChemiDoc Touch Imaging System running Image Lab 5.2.1 software (Bio-Rad). After being stripped with mild stripping buffer, the membrane was reprobed with a β-actin antibody (2 µg/mL, ab6276 monoclonal mouse, Abcam; RRID:AB_2223210) to normalize for gel loading.

### Computational Predictions of Human Radial Artery Shear Stress

As NO production influences vascular remodeling, shear stress assays with an NO sensor dye were conducted using a human microvascular endothelial cell line. These assays aimed to reflect the shear stress that a human radial artery would be exposed to in vivo. First, the radial artery shear stress rates in different physiological and pathological scenarios across the first half of human pregnancy were predicted using a computational model of the human uteroplacental artery network, parameterized to reflect physiological scenarios at 6–8, 10–12, or 16–20 wk of gestation, including physiological measurements of trophoblast plugs and channels previously reported (7).

Allerkamp et al. (7) quantified the anatomical progression of spiral artery remodeling across the first half of pregnancy. The spiral arteries have classically been described mathematically as funnel-like vessels (17, 38) or tubes blocked with a porous trophoblast plug (14). More recent anatomical descriptions showed an evolving channel forming in these plugs over gestation (7, 13). Here, we describe the resistance of spiral arteries as a plugged tubular structure at 6–8 wk of gestation, with an increasing funnel-like shape and channel structures forming in trophoblast plugs as gestation progresses.

The anatomy described in the model is based on a previously published model of the uterine circulation at term (10), combined with mathematical descriptions of plugs in the spiral arteries early in gestation (14). The arterial vessel network feeding the intervillous space of the placenta is defined by level (generation) with level *i* representing *n_i_* anatomically defined vessels (*i* = 1 represents the uterine artery, *i =* 2 the arcuate arteries, *i =* 3 the radial arteries, *i =* 4 arteriovenous anastomoses, *i* = 5 the spiral arteries, and *i =* 6 the intervillous space). For all vessels that are not plugged by trophoblast (uterine, arcuate, and radial arteries, arteriovenous anastomoses) the vessel segment resistance (*R*) is defined as a Poiseuille resistance (14). Resistance of the intervillous space is assumed constant (10, 39). The spiral arteries are divided into segments, where the total resistance of a spiral artery is defined as the sum of the resistance of each segment. In early gestation, there are “tube-like” segments where there is no trophoblast plugging and change in radius over the length of the segment is minimal. These segments are assumed to have a Poiseuille resistance. In addition, we define the resistance for funnel-like segments (where there is no trophoblast plugging, but the radius of the vessel increases along its length), fully plugged segments (where the artery is filled with a plug of trophoblast cells), and plug and channel segments (where a channel has formed in the center of the plug).

#### Funnel-like segments.

The resistance of funnel-like segments follows Burton et al. (17) and is defined by

(*1*) $$R\;=\;\frac{8\;}{\pi r_{a}^{4}}\left(\frac{r_{a}}{3c}-\frac{r_{a}^{4}}{3c{\left(r_{a}+cL\right)}^{3}}\right)\;,$$

where μ is blood viscosity, *L* is the length of the funneled section, *r_a_* is the radius of the vessel at the start of the funnel, *c* = (*r_b_* − *r_a_*)/L, and *r_b_* is the (larger) radius of the vessel at the end of the funnelled section.

#### Fully plugged segments.

Plugged spiral arteries are modeled, following James et al. (14), using the Darcy–Brinkman law:

(*2*) $$R\;=\;\frac{\;L}{K\pi r^{2}}\left(1-2\left(\frac{\left(\sqrt{\frac{K}{\gamma }}\right)}{r}\frac{I_{1}\left(\sqrt{\frac{\gamma }{K}}r\right)}{I_{0}\left(\sqrt{\frac{\gamma }{K}}r\right)}\right)\right)\;,$$

where *I*_0_ and *I*_1_ are modified Bessel functions, μ is blood viscosity, *L* is length of arterial section, *r* is radius, *K* is permeability of the plug, and γ is an effective viscosity. Both *K* and γ are determined by the porosity of the plug (Φ) and is defined as the ratio of total volume of void space to total volume of trophoblast cells in the plug. Permeability (*K*) was estimated using Carman–Kozney relationship (40), and $\gamma \;=\;\frac{1}{\left[1\;+\;2.5\left(1\;-\;\phi \right)\right]}$ (40, 41).

#### Plug and channel segments.

Plugs with channels have been previously modeled using a spatially varying porosity (42) in *Eq. 2*. However, following similar analyses by Khaled and Vafai (43), here an analytical expression for resistance of a plugged region with a channel in its center. The expression for resistance in these sections is defined in the appendix, with resistance (*R*) being a function of plug porosity, as well as the relative radii of the channel and the plug that surrounds it.

With the use of the resistance of each vessel level and the connectivity of vessels, a distribution of blood flow rate and shear stress is calculated within the uterine circulation feeding the placenta using electric circuit theory (14, 43). This theory allows the resistance at each vessel level to be summed in parallel, with total resistance at each level summed in series. This is the case with all vessels except arteriovenous anastomoses, which arise in parallel to the spiral arteries feeding the placental bed (10). With a resistance for the uterine circulation feeding the placenta, a constant “myometrial resistance” is defined to represent the uterine circulation that does not feed the placenta [see the study by Talbert et al. (39)], which is added in parallel to produce a total uterine vascular resistance. Myometrial resistance is assumed to be unchanged over the course of gestation, with the uterine arterial remodeling in arteries feeding the placenta dominating.

Boundary conditions for the model are defined at the uterine artery. Mean uterine artery flow and pressure typical of each gestational stage is defined from the literature (17, 44, 45) (appendix Table A1) and flow division through each level of the vascular tree network can then be estimated. Finally, shear stress, τ, on the endothelial cells in the radial arteries is defined as

$$\tau \;=\;\frac{4\mu Q}{\pi r^{3}}.$$

Parameterization of the model is given in appendix Table A1, and the code used to run the models is available: https://github.com/VirtualPregnancy/placenta-simulations.

**Figure 1.**
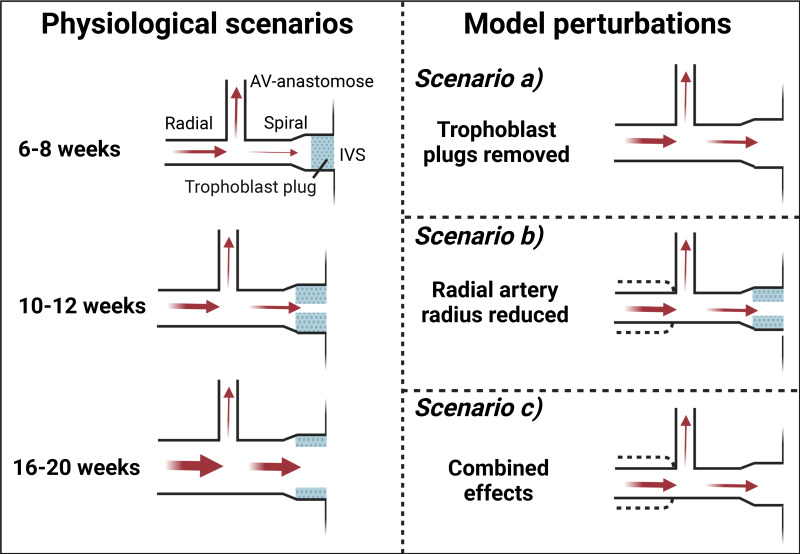
Illustration of computational model of the human uteroplacental artery network. Schematic showing the modeled physiological scenarios at each time point (*left*) and the model perturbations (*right*) that were used for each time point, respectively. AV image was created with a licensed version of BioRender.com.

In addition to modeling the physiological anatomy of the arteries at 6–8, 10–12, and 16–20 wk, we perturbed the model to reflect where different aspects of physiological remodeling are impaired (formation of trophoblast plugs, widening of the radial arteries, plug break down) (2, 42). We *1*) removed trophoblast plugs in the spiral arteries, *2*) reduced the diameter of the radial arteries, and *3*) considered the combined effects of *1* and *2*, relative to the normal physiological scenario by both reducing radial artery diameter to 85% and removing the plug (Fig. 1). The resulting predicted rates of shear stress in the upstream radial arteries were compared with the physiological scenarios. To cover a broad range of different scenarios that had to lie within the limitations of the assay, shear stress rates of 0, 7, 12, 16, and 20 dyn/cm^2^ were chosen for the final NO tracking experiments.

### Endothelial Nitric Oxide Production Under Shear Stress

HMEC-1 cells (a human microvascular endothelial cell line; RRID:CVCL_0307) were cultured in Endothelial Cell Growth Media (ECGM, CCM027, R&D systems, Minneapolis, MN) containing 1% penicillin-streptomycin and used in shear stress assays at *passages 14–17*. Five days before each shear stress experiment, the three paracrine factors that had shown an effect on vascular reactivity in pressure myography experiments (β-estradiol, progesterone, and PlGF) were added alone or in combination to HMEC-1 in T25 cell-culture flasks. Concentrations were chosen according to those reported in maternal serum in the first half of pregnancy (Table 1) to mimic the physiological conditions for endothelial cells during the most pronounced time of radial artery remodeling (5, 7). The same concentrations were added to the respective cells during all subsequent steps of the experiment including the imaging period. Of note, while VEGF was not investigated separately, ECGM itself contains VEGF of unknown concentration. For shear stress assays, the 48-well setup in the BioFlux200 flow system (Fluxion, San Francisco, CA) was used. After channels were coated with 20 mg/mL of fibronectin (Merck, Darmstadt, Germany), HMEC-1 cells were seeded as previously described (46) and left to adhere at 37°C, 5% CO_2_ in the supplemented ECGM with or without the additional paracrine factor(s) overnight. One confluent T25 flask was used to seed one channel for each of the four shear stress conditions. The next day, seeded cells were incubated with 10 µM diaminofluorescein diacetate (DAF-2DA) (Abcam) in PSS by flushing the channels every 20 min at 2 dyn/cm^2^ for 1 min with fresh PSS + DAF-2DA. After 1 h, channels were washed with PSS containing 90 µM l-arginine (Merck) without DAF-2DA for 10 min at 2 dyn/cm^2^ and then held under static conditions for 30 min before imaging. For each paracrine condition (untreated control, β-estradiol, progesterone, PlGF, combined factors), one shear stress condition per channel (7, 12, 16, or 20 dyn/cm^2^) was applied for the maximum time possible in the Bioflux (45 min for 7 and 12 dyn/cm^2^, 33 min for 16 dyn/cm^2^, 27 min for 20 dyn/cm^2^), as increased shear conditions depleted media in the inlet wells at greater rates. Data were compared for different treatments within each shear stress condition. Two positions in each channel were imaged at ×20 magnification both immediately before the onset of flow (baseline at 0 dyn/cm^2^) and then every 3 min during the flow protocol, using a Nikon TE2000E inverted fluorescence microscope with a Digital Sight DS-Ri2 CMOS sensor color camera running NIS Elements AR software (Nikon Instruments, Melville, NY). This setup was repeated in *n* = 3 or 4 biological replicates. The fluorescence intensity was quantified in each acquired image, which directly relates to intracellular NO production (43). To do this, ImageJ I.50i (NIH) was used to create an overlay of the total area covered by cells by using the “triangle” auto-threshold function for each image. This overlay was analyzed with the “analyze particle” function to measure the mean gray value (MGV) and area of each particle (*A*_part_). Mean gray values were corrected for background fluorescence by subtracting an averaged background measurement of each imaged position resulting in the corrected MGV (MGV_corr_). To achieve a MGV_corr_ that accounts for the size of each particle (MGV_area_), the following was calculated for each particle: MGV_corr_ × *A*_part_ = MGV_area_. To provide a measurement for the mean fluorescence intensity per area for each imaged position at each time point *i* [MGV_timepoint__(_*_i_*_)_], the following was calculated for each image: ∑MGV_area_/∑*A*_part_ = MGV_timepoint__(_*_i_*_)_). To allow a relative comparison between the different imaged positions and conditions (MGV_rel_), for each time point the following was calculated: MGV_timepoint__(_*_i_*_)_/MGV_timepoint(0)_ = MGV_rel_, where *time point 0* is before onset of flow. MGV_rel_ values for both imaged positions in each channel were averaged as technical replicates. The averaged MGV_rel_ values were used for analysis. The workflow is shown in Supplemental Fig. S1 (see https://doi.org/10.6084/m9.figshare.22498207).

### Statistical Analysis

All statistical analyses were conducted using GraphPad Prism (v8.4.2, GraphPad Software). All data were tested for normal distribution using the Shapiro–Wilk test. Quantification of staining intensity of immunohistochemistry experiments was compared between the pregnant and nonpregnant group by Mann–Whitney *U* test. Vessel sizes shown for the myography experiments are expressed as proportional size of their inner diameter preflow. The correlation between the absolute vessel diameter and the proportional diameter change was assessed by Spearman correlation. As this was not significant, relative vessel diameter change across all flow steps between groups were compared using a repeated-measures two-way ANOVA with the flow steps acting as repeated factor, followed by Tukey’s multiple comparison post hoc test. Vessel size at 40-mmHg pressure gradient (maximum constriction of nonpregnant control group) was compared between groups by one-way ANOVA followed by Sidak’s multiple comparison post hoc test. eNOS protein expression in Western blots was compared relative to β-actin, with pooled samples of nonpregnant versus pregnant animals compared by Mann–Whitney *U* test. NO production under shear stress in HMEC-1 cells was compared by calculating the area under the curve for each condition over time and comparing it by one-way ANOVA followed by Dunnett’s multiple comparison post hoc test. *P* values < 0.05 were considered statistically significant.

## RESULTS

### Receptors for Pregnancy-Specific Paracrine Factors Are Expressed in Rat Radial Arteries

**Figure 2.**
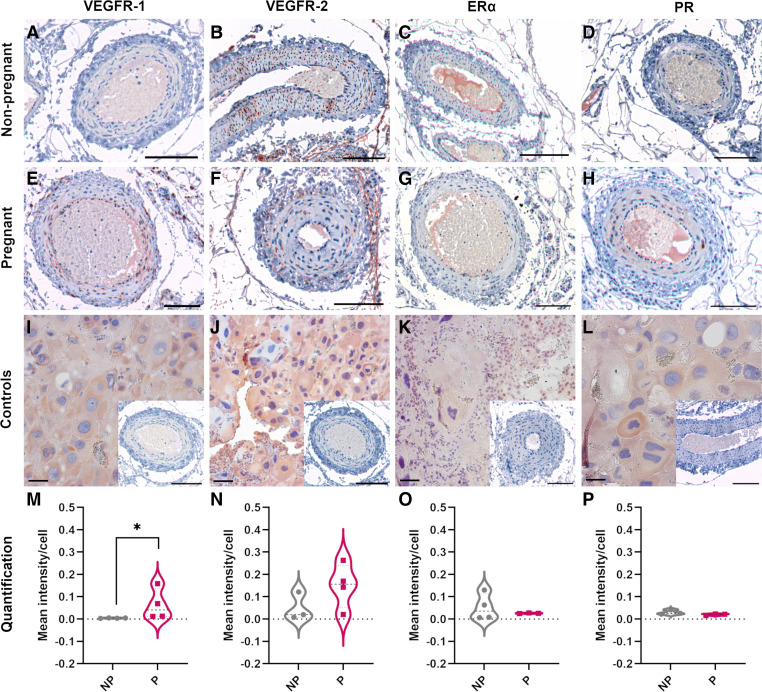
Expression of vascular endothelial growth factor receptor (VEGFR)-1, VEGFR-2, estrogen receptor-α (ERα), and progesterone receptor (PR) in rat radial arteries. Representative images of immunohistochemical staining of nonpregnant (*A–D*) or pregnant (*E–H*) rat radial arteries stained for VEGFR-1 (*A* and *E*), VEGFR-2 (*B* and *F*), ERα (*C* and *G*), or PR (*D* and *H*). Rat placental tissue was used as positive controls (*I–L*), with irrelevant IgG on radial arteries used as negative controls (*I–L*, *insets*). Scale bars = 100 µm. Violin plots show quantification of the staining intensity of VEGFR-1 (*M*), VEGFR-2 (*N*), ERα (*O*), and PR (*P*) within all three layers (tunica intima, media, adventitia) of the radial artery walls of nonpregnant (NP) and pregnant (P) animals. *n* = 3 or 4/group; Mann–Whitney *U* test. *P < 0.05.

To localize the expression of receptors for the paracrine factors investigated in this work (VEGFR-1 and -2, ERα, PR) in rat radial arteries and to consider changes in expression with pregnancy, we undertook immunohistochemistry in tissue sections containing radial arteries from pregnant (E19.5, *n* = 3 or 4) and nonpregnant (*n* = 3 or 4) rats. All four receptors were detected in vascular smooth muscle cells (VSMCs) of the radial arteries and in positive control placental tissue (Fig. 2). VEGFR-1 staining was weak in the nonpregnant group in all three radial artery layers [tunica intima (endothelium), media, and adventitia] but significantly increased in intensity with pregnancy, with the strongest staining observed in the endothelium and adventitia (Fig. 2, *A*, *E*, *I*, and M; *P* = 0.03). VEGFR-2 staining was also seen in all three artery layers but showed no significant differences in mean staining intensity between nonpregnant and pregnant radial arteries. Strong staining for VEGFR-2 was observed in the stromal cells of the neighboring connective tissue in both pregnant and nonpregnant animals (Fig. 2, *B*, *F*, *J*, and N). The immunoreactivity of ERα in the rat radial arteries and surrounding connective tissue was weak. In both nonpregnant and pregnant uterine tissue, infrequent nuclear staining was observed primarily in the outermost tunica adventitia of the vessel and neighboring stromal cells in the connective tissue, with no difference in expression frequency or intensity between the groups (Fig. 2, *C*, *G*, *K*, and *O*). Immunostaining for PR was observed infrequently in the cytoplasm and nuclei in all three radial artery layers in both nonpregnant and pregnant tissue, with no significant difference in the mean staining intensity between groups (Fig. 2, *D*, *H*, *L*, and *P*).

### Pregnancy-Specific Paracrine Factors Attenuate Flow-Mediated Vasoconstriction of Rat Radial Arteries

**Figure 3.**
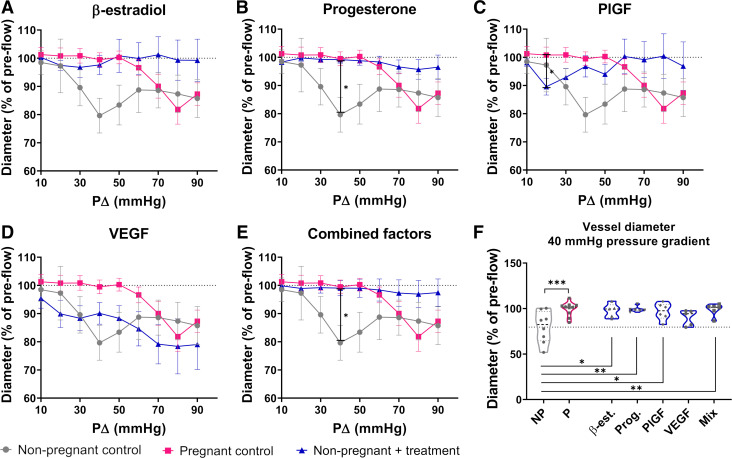
Rat radial artery response to increasing flow rates in pressure myography. *A–E*: graphs showing the change of inner diameter normalized to size before onset of flow across the intramural pressure gradient range (*P*Δ) as acquired in myography experiments. Data of arteries from nonpregnant control animals (*n* = 8, pink) and from pregnant animals (*n* = 10, gray) are taken from Allerkamp et al. (1). Blue lines show treatment groups acquired in this work (nonpregnant radial arteries treated with a single paracrine factor, or combination of all 4; *n* = 5 or 6 per condition). Means ± SE. Two-way ANOVA + Tukey’s multiple comparison post hoc test. *P < 0.05. *F*: violin plots showing inner diameter (normalized to size before onset of flow) of all groups at 40-mmHg pressure drop. Dotted line shows mean size of untreated nonpregnant radial arteries. NP, untreated nonpregnant control; P, pregnant control; β-est, β-estradiol; Prog, progesterone; PlGF, placental growth factor; VEGF, vascular endothelial growth factor; Mix, combined factors. One-way ANOVA + Sidak’s multiple comparison post hoc test; *n* = 5–10/group. *P < 0.05, **P < 0.01, and ***P < 0.001.

We have previously published data describing the vascular reactivity of untreated radial arteries from nonpregnant and pregnant animals under flow, which was used to develop a computational model of radial artery vascular reactivity (32). As this prior work was undertaken in the exact setup used here, data are directly comparable as baseline values. As reported, the predominant response to flow in radial arteries from both nonpregnant and pregnant animals was vasoconstriction and, importantly, radial arteries constricted only after reaching flow rates compared with those predicted to be physiologically normal [60.4 µL/min pregnant vs. 11.8 µL/min untreated nonpregnant (1)], leading to a maximum constriction at a pressure gradient of 40 mmHg for nonpregnant arteries versus 80 mmHg for pregnant arteries (Fig. 3, *A*–*E*). Here, we show that this maximum flow-mediated constriction of nonpregnant arteries was attenuated by addition of β-estradiol, progesterone, or PlGF, but not by VEGF (Fig. 3). However, only progesterone treatment alone significantly reduced the maximum constriction (inner diameter of nonpregnant controls at 40 mmHg relative to preflow was 79.6 ± 17.4%, compared with 99.2 ± 3.1% in nonpregnant vessels after progesterone treatment (*P* = 0.04; Fig. 3, *B–E*). Combined treatment with β-estradiol, progesterone, PlGF, and VEGF also significantly reduced the maximum constriction (inner diameter at 40 mmHg after combined paracrine treatment was 99.0 ± 6.6%, compared with 79.6 ± 17.4% in untreated nonpregnant controls (*P* = 0.04; Fig. 3*E*). When the radial artery diameter specifically at the maximum constriction level of untreated nonpregnant vessels (40 mmHg) was compared between groups, this was significantly larger in the pregnant compared with the untreated nonpregnant group (*P* = 0.001). Treatment of nonpregnant radial arteries with β-estradiol, progesterone, PlGF, or all paracrine factors combined (but not with VEGF alone) led to a significant increase in artery diameter (*P* < 0.02; Fig. 3*F*), similar to that seen in the pregnant state. Likewise, treatment with β-estradiol, progesterone, PlGF, or all paracrine factors combined enabled nonpregnant arteries to withstand even higher flow rates than the pregnant control group showing maximum constriction at a pressure gradient of 80 mmHg (Fig. 3, *A*–*C* and *E*).

### Attenuation of Flow-Mediated Vasoconstriction by Paracrine Factors Is Partially Mediated by eNOS

To test whether the attenuation of vasoconstriction in response to flow observed in the presence of paracrine factors was mediated by eNOS, nonpregnant arteries were incubated with the eNOS antagonist l-NAME before incubation with β-estradiol, progesterone, PlGF, or the combination of all three of these factors with VEGF. As VEGF alone did not show an effect on nonpregnant radial artery reactivity (Fig. 3, *D* and *F*), data on VEGF with l-NAME are not shown.

**Figure 4.**
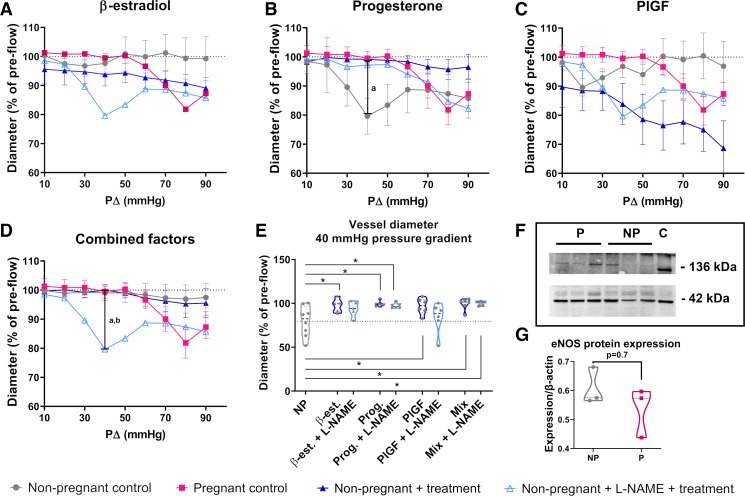
Influence of endothelial nitric oxide synthase (eNOS) on rat radial artery response to increasing flow rates after treatment with paracrine factors. *A–D*: graphs showing the change of inner diameter normalized to size before onset of flow across the intramural pressure drop range as acquired in myography experiments using nonpregnant (*n* = 8, gray) and pregnant (*n* = 10, pink) control arteries [data taken from Allerkamp et al. (1)], nonpregnant arteries treated with single/combined paracrine factors (*n* = 5 or 6, dark blue), or nonpregnant arteries treated with *N*^ω^-nitro-l-arginine methyl ester (l-NAME) before single/combined paracrine factors (*n* = 5 or 6, light blue). Means ± SE; two-way ANOVA + Tukey’s multiple comparison post hoc test. a = *P* < 0.05 for nonpregnant vs. pregnant control; b = *P* < 0.05 for nonpregnant control vs. nonpregnant + treatment + *N*^ω^-nitro-l-arginine methyl ester (l-NAME). *E*: violin plots showing inner diameter (normalized to size before onset of flow) of all groups at 40-mmHg pressure drop. Dotted line shows mean size of nonpregnant control group. NP, untreated nonpregnant control; β-est, β-estradiol; Prog, progesterone; PlGF, placental growth factor; Mix, combined factors. One-way ANOVA + Sidak’s multiple comparison post hoc test; *n* = 5–10/group. *P < 0.05. *F*: Western blot showing pregnant (P) and nonpregnant (NP) rat radial artery lysate samples and rat placental lysate control (*C*). eNOS bands were detected at a molecular weight of 136 kDA. β-Actin (42 kDa) was used as loading control. *G*: violin plot showing quantification of Western blot analysis of eNOS protein expression in radial artery tissue lysates relative to β-actin expression; *n* = 3 pools of vessels from *n* = 9 nonpregnant rats (NP), *n* = 3 pools of vessels from *n* = 9 pregnant rats (P), run in two technical replicates; Mann–Whitney *U* test.

For both β-estradiol and progesterone, concurrent l-NAME treatment shifted radial artery diameters from the pregnant baseline toward the nonpregnant baseline, suggesting that induction of eNOS by both factors is partly, but not completely, involved in attenuating the constrictive response to flow (Fig. 4, *A* and *B*). In contrast, l-NAME was able to completely remove the effect of PlGF in attenuating constriction, resulting in more pronounced flow-mediated constriction than in the nonpregnant controls and suggesting a dominant role of eNOS downstream of PlGF in attenuating the constrictive response to flow (Fig. 4*C*). However, l-NAME was not able to alter the vasoactive response when arteries were treated with all four paracrine factors combined (Fig. 4*D*). At 40-mmHg pressure difference (when maximum constriction of untreated nonpregnant vessels occurs), vessels pretreated with l-NAME plus progesterone, or with all four paracrine factors combined, remained significantly larger than untreated nonpregnant control vessels (*P* = 0.03; *P* = 0.01; Fig. 4*E*). This shows that induction of eNOS by combined paracrine factors, as would occur in the uterine microenvironment, only partially attenuates the constrictive response to flow.

As the aforementioned results suggest that single paracrine factors (PlGF, β-estradiol, progesterone) can trigger radial artery adaptation in pregnancy partially through increased NO production by eNOS, we then sought to examine whether this is a result of increased eNOS expression by Western blot. There was no significant difference in eNOS protein expression between untreated radial arteries from nonpregnant and pregnant animals (*P* = 0.7; Fig. 4, *F* and *G*).

### Artery Diameter, Trophoblast Plugs, and Channel Size Affect Shear Stress Rates in Human Uterine Radial Arteries

As human uterine radial arteries are not accessible during pregnancy for ethical reasons, we aimed to translate the aforementioned findings from rat radial arteries to the human scenario using in vitro shear stress cell assays. To do this, first we developed a computational model reflecting the human vascular anatomy across the first half of pregnancy, including trophoblast plugs and the progressive formation of channels within these plugs. This allowed prediction of shear-stress rates expected to act on the endothelium of human uterine radial arteries in physiological scenarios at different time points across the first half of pregnancy, i.e., when the radial arteries are remodeling (Table 2).

**Table 2.** Shear stress predictions in human uterine radial arteries

	Gestation		
Spiral Arteries	6–8 wk	10–12 wk	16–20 wk
Physiological size, dyn/cm^2^			
Plugged	6.4*	12.0*	28.4*
Not plugged	10.6	39.8	29.3
85% of physiological size, dyn/cm^2^			
Plugged	10.3	19.3	45.7
Not plugged	17.0	62.8	47.2

* Values for the physiological scenario at each time point.

**Figure 5.**
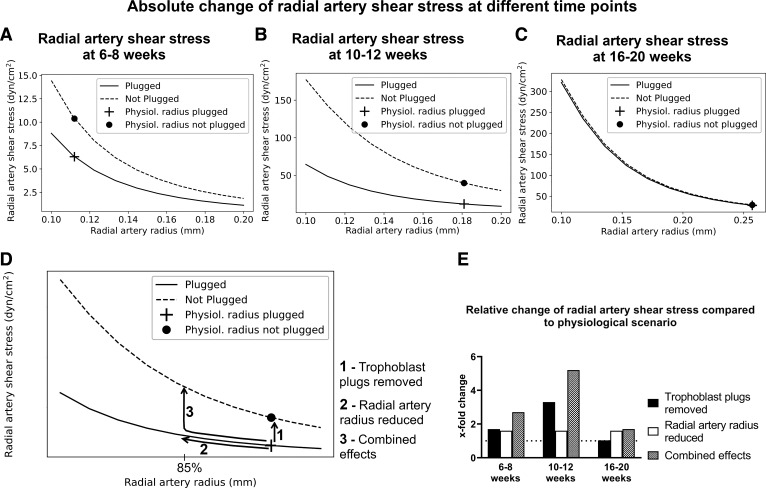
Model predictions of change of shear stress within human radial arteries in different scenarios. *A*–*C*: relationship between radial artery radius and shear stress in a plugged (solid line) or unplugged (dotted line) scenario at 6–8 (*A*), 10–12 (*B*), and 16–20 (*C*) wk of pregnancy. The cross and dot mark the physiological radial artery radius in each case. *D*: schematic of the model perturbations to reflect pathological scenarios. Removing the plug from the spiral arteries is labeled with *process 1*, reducing radial artery radius to 85% with *process 2*, and the combined effects with *process 3*. *E*: bar graphs showing the *x*-fold change of shear stress compared with the physiological scenario at 6–8, 10–12, and 16–20 wk of gestation when either *1*) plugs are removed (*scenario a*), *2*) radial artery diameter was reduced to 85% of its physiological value (*scenario b*), or *3*) the combination of both (*scenario c*).

To assess the aspects of the uterine circulation that have the largest impact on shear stress within the radial arteries across the first half of pregnancy, we ran the model in a range of scenarios to model impaired trophoblast plugging and/or impaired radial artery remodeling. Mimicking premature unplugging by removing trophoblast plugs, while holding other parameters at physiological levels (appendix Table A1), resulted in predictions of increased shear stress in the radial arteries, increasing 1.7-fold at 6–8 wk of gestation, and 3.3-fold at 10–12 wk of gestation, with negligible increase at 16–20 wk when physiological plugs take up a smaller proportion of the vessel lumen (Fig. 5, *A*–*C*, change from cross to dot; Table 2; Fig. 5*D*, *arrow 1*, and Fig. 5*E*). Layering on top of this, we further assessed the impact of increased channel size in trophoblast plugs (i.e., demonstrating a more physiological degree of accelerated breakdown, rather than their complete removal) at 10–12 wk of gestation. In this scenario, a 1.6-fold increase of channel size (from 0.034 to 0.055 mm) was predicted to double the shear stress rate from 12 to 23.8 dyn/cm^2^ within the radial arteries.

When impaired radial artery remodeling was simulated by reducing the radial artery radius (with all other parameters held at physiological levels), shear stress within the radial arteries was predicted to exponentially increase at all simulated time points (Fig. 5, *A*–*C*, solid line). Decreasing the diameter to 85% of the physiological size was predicted to increase shear stress by 1.6-fold at each time point (Table 2; Fig. 5*D*, *arrow 2*, and Fig. 5*E*). To consider how these effects interact, we then simulated the impact of a lack of plugging when the radial artery radius was also reduced to 85% (Fig. 5*D*, *arrow 3*). In this scenario, the model predicts the most pronounced change in radial artery shear stress at all predicted gestational time points (Fig. 5*E*). Shear stress was predicted to increase by 2.7-fold at 6–8 wk of gestation, 5.2-fold at 10–12 wk of gestation, and 1.7-fold at 16–20 wk of gestation compared with normal physiological scenarios (Table 2; Fig. 5*E*). In contrast to the 6–8 and 10–12-wk scenarios, the predicted shear stresses in the plugged and unplugged scenarios almost completely overlap at 16–20 wk of gestation (Fig. 5*C*), showing that plugs do not have an impact on radial artery shear stress at this time independently of radial artery size.

### Paracrine Factors Increase NO Production in HMEC-1 Cells Under Relevant Shear Stress

We next sought to determine the impact of the changes in radial artery shear stress predicted computationally in the aforementioned scenarios on human microvascular endothelial-cell (HMEC-1) NO production in the presence or absence of paracrine factors, allowing us to better understand the interaction of paracrine and hemodynamic factors on radial artery remodeling. To do this, HMEC-1 cells were treated with either PlGF, β-estradiol, progesterone, or a combination of all three and the relative fluorescence intensity over time tracked under 7, 12, 16, or 20 dyn/cm^2^ of shear stress, chosen to cover a range of shear stress scenarios within the radial arteries across the first half of pregnancy (Table 2). Here, 7 dyn/cm^2^ reflects the physiological scenario at 6–8 wk of gestation, 12 dyn/cm^2^ reflects the physiological scenario at 10–12 wk of gestation, and 16 and 20 dyn/cm^2^ reflect the physiological scenario when progressing to 16–20 wk of gestation, as well as scenarios of impaired remodeling up to 12 wk of gestation.

**Figure 6.**
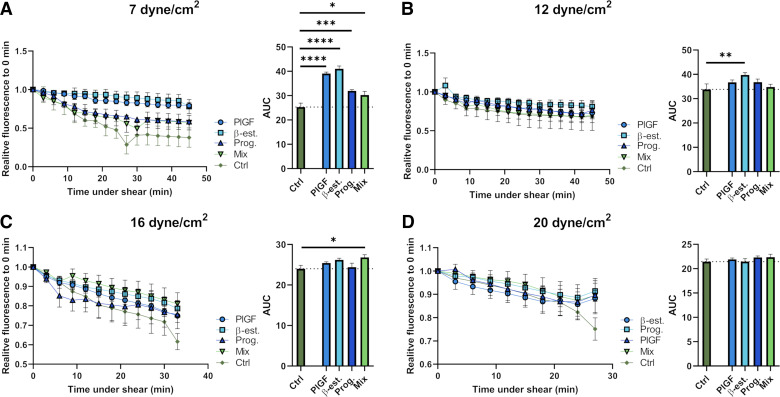
Influence of paracrine factors on nitric oxide (NO) production in human microvascular endothelial-cell (HMEC)-1 cells under different shear stress. Graphs showing the relative fluorescence of HMEC-1 cells under shear stress of 7 (*A*), 12 (*B*), 16 (*C*), or 20 (*D*) dyn/cm^2^ over time for controls (Ctrl) or HMEC-1s treated with placental growth factor (PlGF), β-estradiol (β-est), progesterone (Prog), or the combination of all factors (Mix). Bar graphs showing the area under the curve (AUC) calculated for each condition are presented alongside. Data are from *n* = 3 or 4 biological replicates/group, run in technical duplicates. AUC was compared by one-way ANOVA + Dunnett’s post hoc test to each nontreated control group. Symbols and bar graphs show means ± SE. *P < 0.05, **P < 0.01, ***P < 0.001, and ****P < 0.0001.

Under all shear stress conditions, NO production was lowest when HMEC-1 cells were not treated with paracrine factors (Fig. 6, *A*–*D*). Under 7 dyn/cm^2^ of shear stress, all treatment conditions induced significantly greater NO production, which was highest with β-estradiol treatment (*P* < 0.0001; Fig. 6*A*). Under 12 dyn/cm^2^ of shear stress, only β-estradiol treatment significantly increased NO production (*P* = 0.01; Fig. 5*B*). Under 16 dyn/cm^2^, only the combination of all paracrine factors significantly increased NO production (*P* = 0.01; Fig. 6*C*). Finally, under 20 dyn/cm^2^, none of the treatments had a significant effect on NO production (Fig. 6*D*).

## DISCUSSION

In this study, we sought to examine the impact of paracrine factors on flow-induced radial artery reactivity and endothelial cell NO production to better understand how the functionally important uterine radial arteries adapt to pregnancy. We show that β-estradiol, progesterone, and PlGF enable radial arteries from nonpregnant rats to withstand higher flow rates compared with the pregnant state and that this effect is most pronounced when all factors are combined. Our results demonstrate that this effect is only partly mediated via eNOS activity and that other flow-sensitive signaling pathways likely contribute. To relate these mechanisms more directly to key structural and hemodynamic changes in the human uterine circulation, we developed a computational model specific to gestational time points across the first half of human pregnancy, incorporating the impacts of physiological trophoblast plugs and impaired radial artery size on uterine hemodynamics. This allowed us to consider how interactions between paracrine factors and shear stress in the human radial arteries may impact endothelial cell NO production, and together suggest a synergistic effect of paracrine and shear stress-mediated vascular remodeling in the first half of pregnancy.

Although most systemic vessels dilate in response to flow, our previous work has demonstrated that rat radial arteries undergo flow-mediated constriction, but in pregnancy adapt to sustain higher flow rates before constricting (1). Here, we aimed to investigate whether paracrine factors, which increase their levels in pregnancy, can induce this tolerance of higher flow rates. Expression of receptors for all paracrine factors used in this work (β-estradiol, progesterone, PlGF, and VEGF) was present in the walls of rat radial arteries, showing their physiological relevance and ability to respond to the paracrine factors present. However, only VEGFR-1 was significantly upregulated in pregnancy, which is consistent with its predominant ligand PlGF being produced by the placenta (and thus only in pregnancy) and is in accordance with prior work (20).

The steroid hormones β-estradiol and progesterone are well-known contributors to blood flow regulation in the female reproductive system throughout the estrus cycle and in pregnancy (47). This is thought to be mediated through both endothelium-dependent vasodilatory effects and direct effects on the vascular smooth muscle cells impacting vascular reactivity and myogenic tone (22, 48, 49). For PlGF and VEGF, distinct effects on the uterine vasculature are less well described, although they are thought to have greater impacts on uterine than systemic vessels (20, 50). Here, we demonstrate for the first time that β-estradiol, progesterone, or PlGF alone, as well as a combination of all factors (including VEGF), reduced the vascular reactivity of nonpregnant radial arteries, enabling them to act more like pregnant vessels and withstand higher flow and shear stress rates.

NO is one of the most potent vasodilators and plays key roles in longer-term vascular remodeling (51). All paracrine factors investigated in this work have been previously shown to induce NO production, albeit by different pathways. Estrogen induces endothelial NO production directly via ERK-mediated phosphorylation of eNOS (18, 21) and indirectly via upregulation of the angiotensin type-II receptor (52). Progesterone can induce eNOS alone but has a greater effect when administered in combination with estrogen (22). PlGF induces vasodilation at least partly via NO, with more pronounced effects reported during pregnancy (20). Finally, VEGF/VEGFR-2 signaling can induce both short- and long-term upregulation of eNOS via various calcium-dependent intracellular pathways (23). Considering this, we used the eNOS antagonist l-NAME to investigate the extent to which eNOS activity was responsible for the paracrine-induced attenuation of flow-mediated constriction observed. This data showed that while l-NAME completely reversed the effect of PlGF and partly reversed the effect of β-estradiol and progesterone alone, it did not affect radial arteries treated with all factors combined. This aligns with earlier work on uterine arteries showing that NO only accounted for ∼70% of estrogen-mediated vasodilation in sheep (53), ∼90% of PlGF-mediated vasodilation in rats, ∼40% PlGF-mediated vasodilation in humans (20), and ∼60% of VEGF-mediated vasodilation in rats (32). That blocking eNOS in our work had only a partial effect on β-estradiol- and progesterone-induced dilation and was not sufficient to impact arteries treated with all factors suggests that other mechanisms mediating the adaptation of vascular reactivity, i.e., other vasodilatory molecules, ion channels, or changes in calcium signaling, are involved in radial artery remodeling (3, 23, 54–57). Finally, to better understand the relative impact of paracrine factors on eNOS expression or activity, we quantified eNOS expression in rat radial arteries by Western blot analysis. That no significant difference in eNOS expression was observed between the pregnant and nonpregnant states, combined with the fact that effects of paracrine factors on vascular reactivity were observed following relatively short-term incubations, suggests that the primary mechanism of action may be on eNOS activity rather than total eNOS expression levels.

Human uterine radial arteries cannot be accessed during pregnancy for ethical reasons. Therefore, to translate our findings from the rat model to human pregnancy, we investigated the effects of paracrine factors and shear stress at the cellular level by combining the use of human microvascular endothelial cells (HMEC-1) with computational predictions of shear stress rates in human uterine radial arteries in different physiologically relevant scenarios across the first half of pregnancy. Our modeling results predict that absent trophoblast plugs, or trophoblast plugs with larger channels than observed physiologically, increase radial artery shear stress until 10–12 wk of gestation, while their much-reduced occupation of the arterial lumen at 16–20 wk of gestation means their influence on radial artery hemodynamics becomes negligible. If radial arteries do not remodel effectively and remain smaller than normal, as has been hypothesized to occur in FGR (10), a consistent increase in shear stress is predicted at all gestational time points modeled. The combination of inadequate plugging and insufficient remodeling of the radial arteries is predicted to lead to the largest increase in radial artery shear stress at all time points. However, this evoked the most prominent shear stress increase at 10–12 wk of gestation, primarily due to the influence of absent trophoblast plugs. This highlights the potential importance of structural changes in trophoblast plugs on upstream hemodynamics in the system at the time when the most pronounced remodeling of the radial arteries is beginning (7). Although our modeling data show that radial artery shear stress increases physiologically across gestation, our pressure myography data suggest that the influence of β-estradiol, progesterone, and PlGF is needed to enable the arteries to withstand those increasing flow rates without constricting. Subsequently, supraphysiological radial artery shear stress due to inadequate trophoblast plugging and/or impaired outward radial artery remodeling in FGR could plausibly lead to flow-mediated constriction of the radial arteries and thereby further limit blood flow to the placenta.

The in silico data, as well as the pressure myography experiments, demonstrate the synergistic importance of both hemodynamic and paracrine effects underlying radial artery remodeling. We thus further explored the interaction between hemodynamic and paracrine factors in vitro by examining their influences on endothelial NO production. This showed that overall, paracrine factors enhanced endothelial NO production under 7–16 dyn/cm^2^ shear stress conditions. However, variations in the impact of paracrine factors at different shear stress levels let us consider how this interaction may play out in vivo across the first half of pregnancy. In very early pregnancy (6–8 wk of gestation) when trophoblast plugs in the spiral arteries are very compact and radial artery shear stress predictions are ∼ 7 dyn/cm^2^, our in vitro data show that NO production can be increased by individual paracrine factors. This suggests that initially NO production in the radial arteries is predominantly a response to paracrine cues from β-estradiol, progesterone, and PlGF. As shown by our pressure myography data, these cues also enable them to withstand the increasing flow. However, at the time of most pronounced changes in radial artery diameter [12 to 16 wk of gestation (7)] when higher radial artery shear stress rates (≥12 dyn/cm^2^) are predicted, paracrine factors have a less dominant impact on NO production, with increased endothelial NO production only evident when all paracrine factors were combined at 16 dyn/cm^2^. Together, this shows that while paracrine factors play important roles in NO-driven remodeling of the radial arteries under lower shear conditions in the first trimester, as blood flow and shear stress increase across gestation, hemodynamics begin to have a more dominant impact on radial artery NO production and corresponding reactivity.

Our in silico model of the uterine circulation is based upon theory that has been previously used to model Doppler waveforms throughout gestation (10, 39, 58), as well as models that describe the impact of spiral artery remodeling (14, 17, 42). However, the impact of early gestation spiral artery remodeling has not been previously incorporated into a model of the entire uterine circulation. In addition, we parameterized our model to anatomical data on arterial structure, which has been lacking in previous models of this circulation (10, 14). The model does assume a steady, laminar, and fully developed flow in each artery within the uterine vascular network and that flow profiles are not significantly impacted by vessel tortuosity. Prior analysis of Reynolds, Womersley, and Dean numbers in this system suggest that these assumptions are reasonable, particularly in early gestation (14, 38). In the region of trophoblast plugging, there may be influences of red blood cells on hemodynamic function, and this is neglected in the model presented here. These effects could be twofold: first, in small vessels where non-Newtonian effects due to red blood cells are significant and may influence effective blood viscosity; and second, the effect of red blood cells becoming lodged in plugs or channels. As the diameter of all vessels in our model is much larger than red blood cell diameters, these effects would most likely be in the spiral arteries, and their impact would be a useful target for future simulation studies, as has been considered in the intervillous space (59).

Although we endeavored throughout this work to mimic the physiological environment as closely as possible, this study does have some limitations. First, while animals were used at the same day of the estrus cycle/pregnancy, serum levels of paracrine factors in the animals used were not measured, and thus we were not able to account for potential differences in chronic exposure of the vessels to these factors. Second, as the concentration of paracrine factors in the uterine microenvironment are unknown, concentrations of paracrine factors in the shear stress assays were chosen according to maternal serum levels. However, the placental production of the paracrine factors implies that uterine concentrations of these factors are even higher likely due to venoarterial communication within the uteroplacental vasculature (60), and further studies revealing the composition of the uteroplacental microenvironment would build an important base for comparable experiments in the future. Finally, HMEC-1 cells used in the shear stress assay were chosen because of *1*) the advantages of a cell line over primary cells, *2*) their origin from blood vessels of relevant size, and *3*) the previously shown differential behavior to shear stress (61). However, these are cells of male origin, and female cells would be a more physiologically ideal model.

### Conclusions

In this study, we show that the pregnancy-specific paracrine factors β-estradiol, progesterone, and PlGF are important mediators for the adaptation of rat radial arteries to higher flow rates in pregnancy. While on a cellular level these factors are able to trigger the release of endothelial NO additional to the shear stress-mediated NO production, NO only seems to play a partial role for the adaptation of vessel reactivity. Our results highlight the importance of combined effects of paracrine factors within the uteroplacental microenvironment acting synergistically with the hemodynamic changes to facilitate sufficient radial artery adaptation to pregnancy. This implies that both concentrations of the paracrine factors that are too low, as well as shear stress rates that are too high, may negatively impact the balance between shear stress and paracrine-mediated influences required for successful radial artery remodeling in early pregnancy. We hypothesize that in cases of impaired remodeling, such as is implicated in FGR (5), paracrine cues needed for early remodeling could be missing. This would lead to *1*) insufficient outward remodeling and *2*) insufficient adaptation to increasing flow rates. Together with inadequate trophoblast plugging this would increase the shear stress in the radial arteries, lead to further vasoconstriction, and thereby drive a negative cycle of radial artery adaptation to pregnancy, which could require higher levels of paracrine factors to overcome. Therefore, understanding the mechanistic pathways linking hemodynamic factors and the interplay of paracrine triggers for uterine vascular adaptation has the potential to help better identify and manage FGR babies in which impaired adaptation of the uterine vasculature is implicated in pathology.

## DATA AVAILABILITY

Data will be made available upon reasonable request.

## SUPPLEMENTAL DATA

10.6084/m9.figshare.22498207 Supplemental Fig. S1: https://doi.org/10.6084/m9.figshare.22498207.

## GRANTS

This work was supported by Royal Society of New Zealand Te Apārangi Marsden Fund Grant 18-UOA-135.

## DISCLOSURES

No conflicts of interest, financial or otherwise, are declared by the authors.

## AUTHOR CONTRIBUTIONS

H.H.A., A.R.C., and J.L.J. conceived and designed research; H.H.A., S.C.L., and T.P. performed experiments; H.H.A., S.C.L., and T.P. analyzed data; H.H.A., T.P., and A.R.C. interpreted results of experiments; H.H.A. and S.C.L. prepared figures; H.H.A., A.R.C., and J.L.J. drafted manuscript; H.H.A., A.R.C., and J.L.J. edited and revised manuscript; H.H.A., S.C.L., T.P., A.R.C., and J.L.J. approved final version of manuscript.

## APPENDIX

### Definition of Vessel Resistance in Trophoblast Plugs with a Clear Central Channel

For spiral artery segments with channels, we assume that the channel forms through the center of the segment; that is, the channel spans radius (*r*) over the range 0 ≤ *r* ≤ *r*_1_, and the artery has radius *r*_2_ such that the plug resides within *r*_1_ < *r* ≤ *r*_2_. Under these conditions, flow through the channel, *Q*_1_, is

$$Q_{1}\;=\;\frac{2\pi }{\mu L}\left(c_{2}\frac{r_{1}^{2}}{2}-\frac{r_{1}^{4}}{16}\right),$$

where *L* is the length of the section of the vessel, *r*_1_ the radius of the clear channel, and *c*_2_ is a constant defined by

$$c_{2}\;=\;\frac{1}{2\sqrt{\delta }}\left[\frac{-2k\sqrt{\delta }K_{1}\left(\sqrt{\delta r_{1}}\right)-r_{1}K_{0}\left(\sqrt{\delta r_{2}}\right)I_{0}\left(\sqrt{\delta r_{1}}\right)}{\left(K_{0}\left(\sqrt{\delta r_{2}}\right)I_{1}\left(\sqrt{\delta r_{1}}\right)+K_{1}\left(\sqrt{\delta r_{1}}\right)I_{0}\left(\sqrt{\delta r_{2}}\right)\right)}\right]\;-\frac{1}{2\sqrt{\delta }}\left[\frac{2\sqrt{\delta k}I_{1}\left(\sqrt{\delta r_{1}}\right)-r_{1}I_{0}\left(\sqrt{\delta r_{2}}\right)K_{0}\left(\sqrt{\delta r_{1}}\right)}{\left(K_{0}\left(\sqrt{\delta r_{2}}\right)I_{1}\left(\sqrt{\delta r_{1}}\right)+K_{1}\left(\sqrt{\delta r_{1}}\right)I_{0}\left(\sqrt{\delta r_{2}}\right)\right)}\right]+k+\frac{r_{1}^{2}}{4},\;$$

where δ = γ/*k, I*0, and *I*1 are modified Bessel functions of the first kind, and *K*0 and *K*1 are modified Bessel functions of the second kind.

Fluid flow through the porous surrounds, *Q*_2_, is

$$Q_{2}\;=\;\frac{2\pi }{\mu L}\left(c_{3}\left(r_{2}I_{1}\left(\sqrt{\delta r_{2}}\right)-r_{1}I_{1}\left(\sqrt{\delta r_{1}}\right)\right)-c_{4}\left(r_{2}K_{1}\sqrt{\delta r_{2}}\right)-r_{1}K_{1}\left(\sqrt{\delta r_{1}}\right)+k\frac{r_{2}^{2}-r_{1}^{2}}{2}\right),$$

where *r*_2_ is the radius of the outer tube, and *r*_1_ is the radius of the clear channel. Parameters *c*_3_ and *c*_4_ are constants defined by

$$c_{3}\;=\;\frac{1}{2\sqrt{\delta }}\frac{\left(-2k\sqrt{\delta K_{1}}(\sqrt{\delta r_{1})}-r_{1}K_{0}(\sqrt{\delta r_{2}}\right)}{\left(K_{0}\left(\sqrt{\delta r_{2}}\right)I_{1}\left(\sqrt{\delta r_{1}}\right)+K_{1}\left(\sqrt{\delta r_{1}}\right)I_{0}(\sqrt{\delta r_{2}})\right)},$$

$$c_{4}\;=\;-\frac{1}{2\sqrt{\delta }}\left[\frac{2\sqrt{\delta k}I_{1}\left(\sqrt{\delta r_{1}}\right)-r_{1}K_{0}\left(\sqrt{\delta r_{2}}\right)I_{0}\left(\sqrt{\delta r_{2}}\right)}{\left(K_{0}\left(\sqrt{\delta r_{2}}\right)I_{1}\left(\sqrt{\delta r_{1}}\right)+K_{1}\left(\sqrt{\delta r_{1}}\right)I_{0}\left(\sqrt{\delta r_{2}}\right)\right)}\right].$$

Resistance of the segment, *R*, is defined as

$$R\;=\;\frac{\Delta P}{Q_{1}+Q_{2}},$$

where Δ*P* is the pressure drop across the segment.

Parameterization of the model in different physiological states is shown in Table A1.

**Table A1.** Parameterization of the model in different physiological states

Physiological Value (Reference)			
	6–8 wk	10–12 wk	16–20 wk
Boundary conditions			
Inlet flow, mL/min	24.2^a^	27.2^a^	39.5^a^
MAP, mmHg	80 (45)	82 (45)	84 (45)
Parameter			
Uterine artery			
Number, *n*	1		
Length, mm		100 (10)	
Radius, mm	0.95 (62)	1.18 (62)	1.4 (62)
Arcuate artery			
Number, *n*	2 [estimated from Ref. 63]		
Length, mm	9 [estimated from Ref. 39]		
Radius, mm	0.249^b^	0.32	0.403
Radial artery			
Number, *n*	50 (10)		
Length, mm	6 (10)		
Radius, mm	0.112	0.181	0.257
Arteriovenous anastomoses			
Number, *n*	50 (10)		
Length, mm	6.5 (14)		
Radius, mm	0.032^c^	0.05^c^	0.1^c^
Spiral artery			
Number, *n*	50 [estimated from Refs. 39 and 11]		
Total length, mm	10 [at term; (39)]		
Tube length, mm	7 [estimated from Ref. 17]		
Tube radius, mm	0.036	0.124	0.3
Funnel length, mm	1.7	1.6	3
Funnel radius, mm	0.123	0.175	0.241
Resistance, Pa·s/mm^3^			
IVS	1.3^d^		
Myometrial	25.8^d^		
Plug			
Porosity	0.28	0.123^b^	0.164^b^
Length, mm	1.3	1.4	3
Channel			
Length, mm	No channels	1.4	3
Radius, mm		0.034^b^	0.16^b^

Values were obtained from placenta in situ samples of the Boyd and Dixon collections (University of Cambridge, UK) as previously described (7) if not stated otherwise. IVS, intervillous space; MAP, mean arterial blood pressure.

a Flow rates fit to measurements acquired by Assali et al. (44) and Burton et al. (17); ^b^adjusted measurements acquired from data used in Allerkamp et al. (7); ^c^value fit due to lack of available literature to maintain a physiological pressure drop, and proportion of uterine blood flow directed to the placenta in line with estimates from the literature; ^d^assumed constant, fit to expected flow from ultrasound in a nonpregnant model.
